# Pervasive Digital Twin for PI-Containers: A New Packing Problem

**DOI:** 10.3390/s21237999

**Published:** 2021-11-30

**Authors:** Patrick Charpentier, Frédéric Chaxel, Nicolas Krommenacker, Vincent Bombardier, Fabian Seguel

**Affiliations:** 1 CRAN UMR 7039, Université de Lorraine, CNRS, Campus Sciences, Boulevard des Aiguillettes-BP 70239, 54506 Vandœuvre-lès-Nancy, France; frederic.chaxel@univ-lorraine.fr (F.C.); nicolas.krommenacker@univ-lorraine.fr (N.K.); vincent.bombardier@univ-lorraine.fr (V.B.); 2 Departamento de Ingeniería Eléctrica, Universidad de Santiago de Chile, Avenida Ecuador N° 3519, Estación Central, Santiago 8350709, Chile; fabian.seguelg@usach.cl

**Keywords:** digital twin, packing problem, physical internet

## Abstract

The idea defended in this paper consists in finding, at any time and everywhere, the arrangement of containers within a composite container. The digital image of the real arrangement obtained defines its digital twin. This image evolves at the same time as its real twin. It can be used throughout the logistics chain during loading/unloading phases in hubs, to check the completeness of a load, to find the particular position of a container, etc. This digital twin is obtained through the collection of neighborhood information from the sensor nodes embedded on each container. This embedded solution allows accessibility to this information everywhere. This proximity information and the instrumentation of the containers define new types of constraints and a new version of a packing problem. We propose here a model integrating them. This model is implemented and tested on different test cases, and numerical results are provided. These show that, under certain conditions that will be presented, it is possible to obtain the digital twin of the real arrangement.

## 1. Context and Problem Statement

The problem addressed in this paper consists in establishing a 3D model (or layout) of a real (and therefore existing) composite container in which various parallelepipedic items have been arranged in the physical world. The objective is not only to know the constitution of this arrangement, but above all to find the exact position of the items within the arrangement. The approach deployed to obtain it can be assimilated to an inverse modeling of the Container Loading Problem (CLP) classic problems [1]. The aim here is to establish a model of an existing arrangement, not to establish a model for its implementation. This model must be a permanent reflection of the real system: it is its digital twin. This particular problem appeared us in the Physical Internet (PI) particular context (see [2,3] for more information). One of the important characteristics of the Physical Internet is the encapsulation of goods in standardized modular containers (similar to data packets on the Digital Internet). These containers, called PI-Containers, are then handled, stored, routed, dispatched, etc., by logistic infrastructures (PI-Hubs) organized in the manner of the Internet with its routers [4]. These various and numerous operations (automated or not) on the containers, require rational organization and management. They can only be carried out efficiently if there is a perfect synchronization between the reality of the physical system and that of the information system. The pervasive digital twin of PI-container, that means available anywhere and at anytime, is a potential solution to this synchronization problem.

**Figure 1:**
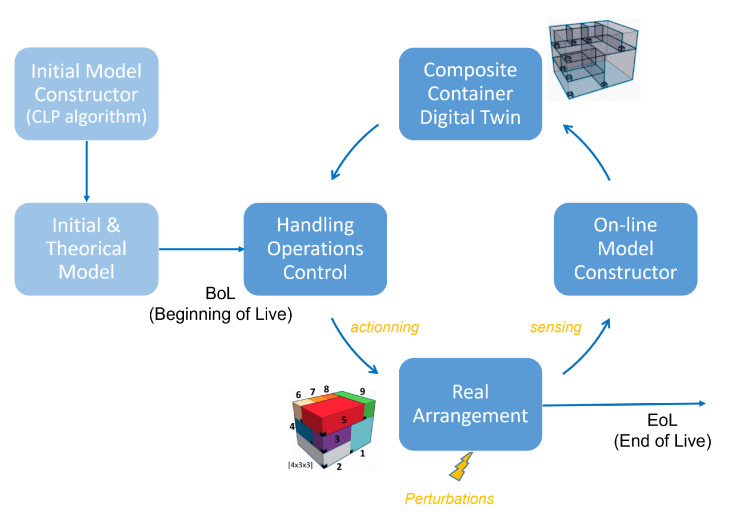
Life cycle of real arrangement and its digital twin.

In this context, the virtualization of containers necessarily involves a step to collect data. To do this, and succinctly (see [5] for more information), we will consider that each container has a wireless sensor network (WSN) node attached to one of its vertices. This node carries at least information about the identifier of the container, its dimensions, and the identifier of the composite in which it is to be inserted. The assembly of different containers within a higher-level container (which we will call “composite container”) makes it possible to constitute an ad-hoc sensors network. The different nodes of this network can then communicate within a certain coverage radius (adjustable parameter of each node by adjusting the radio transmission power) and are able to generate a list of their neighbors (nodes present inside the coverage radius). This information makes it possible to establish a global neighborhood graph (or proximity graph) between all the containers. It will be taken into account by our model. Thus, at any place and at any time, this proximity information may be available and therefore be used to establish the 3D digital twin of the arrangement. Our solution can thus be used for different purposes during the life cycle of the composite container (e.g., full or partial unloading, replenishment, etc.). Figure 1 shows this general process.

The remainder of this paper is organized as follows: Section 2 presents a review of the literature on topics related to our work. Section 3 presents in detail the particular characteristics of the problem treated as well as the proposed mathematical model, and the developed algorithm to solve the model. Section 4 is used to test the model and evaluate the relevance of the approach proposed in this paper. Finally, conclusions and some perspectives to this work are presented in the last section.

## 2. Literature Review

The digital twin (DT) concept was introduced and formalized in 2015 by Michael Grieves in his white paper [6]. A DT is a virtual model of a physical object/system that can be used to simulate the physical state and its behavior in the real space [7]. This has been possible with the advent of sensor, communication network and embedded technologies that enable the twinning process of the physical and the virtual world, through continuous interaction, communication, and synchronization between the physical object and its virtual model [8]. This virtual model presents the advantage of being available remotely and provides value through visualization, analysis, prediction, or optimization [9].

Many of the key enabling technologies are already in place in logistics sectors that conduct to a widespread of digital twins use cases across the entire supply chain. For instance, DT can help to design, operate, or optimize logistics infrastructure like warehouses, distribution centers or cross-dock facilities [10]. In this work, we focus the DT of containers in the Physical Internet paradigm where various and numerous operations will be performed on the composite container during its transportation steps. Unloading of some current items and/or loading of new items will be achieved in hubs, warehouses or cross-dock facilities. In this context, a virtual and computerized counterpart of the physical container could be useful to have at each time an inventory and operational data (size, quantity, location, and characteristics) of every item. This digital twin model (3D layout) could be allowed further of the performance of automation systems such as palletizing/depalletizing handling robots. The object-picking (retrieve one the items within a container) and packing (place the picked-up item in another container) are one of well-known bottlenecks in logistics automation. Solutions are conventionally carried out using simple cameras, industrial 3D cameras, laser triangulation systems, or laser range finders [11]. These conventional viewing systems can only visualize the containers (or parts) placed on the outside (at least partially visible!) of the arrangement. The approach we advocate here is related to the instrumentation of the PI-containers and exploitation of the synchronized DT model corresponding to the composite container.

The digital twin of a composite container can be obtained through the collection of information from the sensor nodes embedded on each container. In this work, we aim to formalize a mathematical model to generate the DT. This problem has many similarities with Cutting and Packing problems (C&P), which can be defined as follows: *“Given a set of components and a set of containers, a packing problem aim to find the set of component positioning variables in order to minimize a set of objectives, while respecting constraints. In all cases, constraints include placement constraints.”* [12]. Indeed, in our problem, we have a set of large objects (here a composite container) and a set of small items (the PI containers) that have been placed within the large object, respecting the two principal placement constraints: all PI containers of the subset lie entirely within the large object, and the PI containers do not overlap. In general, five sub-problems have to be solved simultaneously in order to reach the overall optimum [13]:

- Selection problem regarding the large objects,
- Selection problem regarding the small items,
- Grouping problem regarding the selected small items,
- Allocation problem regarding the assignment of the subsets of the small items to the large objects,
- Layout problem regarding the arrangement of the small items on each of the selected large objects with respect to the geometric condition.

The first four (selection, grouping and allocation) do not concern us since the arrangement is already effective in the composite container. The fifth item is, in its formulation, quite close to our problem. However, we do not seek to “generate” this arrangement, but to “rediscover” it. The objective is the same in both cases and can be formulated as: for each container, how should (or are) the items be arranged? This type of problem is called Single Large Object Placement Problem (SLOPP) in [13], when the layout problem concerns only one arrangement. What has been called the Single Container Loading (Packing) Problem in the literature [14] is an example of the three-dimensional, rectangular SLOPP. It can itself be broken down into two categories: the manufacturer’s pallet loading (MPL) if all boxes are identical, and the distributor’s pallet loading (DPL) when boxes are not of the same size [15].

Our problem belongs to this latter type, the distributor’s pallet loading. However, we are not looking for any optimum (no objective function) and we have to take into account proximity constraints, resulting from measurement performed on the existing arrangement. The proximity constraints mentioned here can, in principle, be assimilated to the positioning constraints mentioned in the literature [1]. These constraints express particular conditions on the positions in which the items are placed in the container. This type of constraint represents the necessity to place all or part of the different items in absolute or relative positions imposed by the problem characteristics [16]. Constraints on the items absolute positioning are linked to particular characteristics of the items (e.g., size, weight or content) which, in certain situations, impose them specific positions (e.g., large or heavy items near the container door to facilitate loading/unloading, dangerous items on the periphery for an easier access) [17]. The relative positioning constraints of items are related to the characteristics of a set of items, either so that they are grouped together (e.g., common delivery of a subset of items, facilitation of completeness verification), or so that, on the contrary, they are distanced (e.g., incompatibility of items, dangerousness) [18,19,20]. The combination of these two types of constraints (absolute and relative) is found in particular in multi-drop situations [20]. They are characterized by the fact that subsets of items go to different customers. The items are thus grouped by destination, but also arranged in the container according to the delivery sequence to avoid unnecessary unloading and reloading operations [21,22]. Two characteristics of the items were defined and used to constrain the positions of the items within the multi-drop framework: visibility and touchable [23]. They guarantee a form of accessibility of the items during deliveries. In the context of our paper, the proximity constraints taken into account are relative positioning constraints of items. They reflect, in a rather abstract way, only the observation of a form of neighborhood or non-neighborhood between items without knowing the cause. They do not have a direct link with the reasons evoked classically in the literature and which we have just briefly presented.

Moreover, each container is considered unique in our case. It has an identifier to distinguish it from other containers of the same dimensions, while usually this is not the case. Finally, the instrumentation of containers generates orientation constraints that are not usually considered in this type of problem. This will be detailed in the following section.

## 3. Problem Statement and Resolution

The nature and the novelty of the problem addressed (generation of a digital twin), as well as the originality of the proximity and orientation constraints, thus pose a new variant of the packing type problems existing to date. The lack of objective defines this kind of problem as a CSP (Constraints Satisfaction Problem). Several potential solutions, satisfying all the constraints, can then appear. Only one of them, nevertheless, corresponds (is compliant) to reality (the real arrangement).

### 3.1. Problem Formulation

The containers considered in this study are of standardized dimensions. Indeed, studies carried out within the framework of Physical Internet [24] have allowed us to define the best container size to maximize space utilization. To free us from the real size of containers, we shall consider here that these are multiple of a standard size sd expressed in meters. Thus, a container i that we will manipulate by its dimensions $\left(a_{i},b_{i},h_{i}\right)\in N^{3}$ will be in the real world of dimensions $\left(sd\times a_{i},sd\times b_{i},sd\times h_{i}\right)$. All the dimensional parameters and variables used in this paper will obey this same principle (e.g., position, radius coverage). This kind of hypothesis is interesting for us, because it reduces the number of potential solutions of 3D arrangement. However, the proposed model would remain valid without this hypothesis. To address the problem, we consider the following definitions:

- Let be a parallelepiped physical composite container of known dimensions ($A_{0}\times B_{0}\times H_{0}$) with an identifier equal to 0. This volume corresponds to the maximum space that the containers (or items) can occupy after being arranged.
- Let *n* be the number of parallelepipedic items (with *id*as unique identifier, id∈[1,n]) of known dimensions $(a_{id},b_{id}\;\mathrm{and}\;h_{id}$ stacked in the composite container. We therefore start here from the assumption that the number n of containers is a *priori* known. It is in fact obtained via the instrumentation of the containers, via a counting algorithm.
- Let $R_{1}$ et $R_{2}$ be transmission ranges (low and high threshold level to take into account an uncertainty related to proximity measurement - if $R_{1}=R_{2}$, uncertainty does not exist, otherwise $R_{2}$ is greater than $R_{1}$). They make it possible to estimate the proximity (or not) of the containers to each other from a radio signal emitted/received by the associated nodes. This notion of proximity is less rich than the distance between nodes estimation (which is obtained by measuring the characteristics of the signal, e.g., power). However, this technique has the advantage of being less sensitive to environmental disturbances. This is sufficient to obtain the neighborhood graph and to represent the proximity relationship among the containers. The notion of proximity between two containers is symmetrical (the graph is non-oriented). In reality, it is not always so, because it is built on the basis of radio exchanges where some disturbances can occur on the measurements made. We shall nevertheless take the hypothesis of symmetry here; our model makes it possible to partially take into account an uncertainty related to the measurement.
- Let G{V,E} be a non-oriented graph (obtained from data collected through PI-containers instrumentation) where:
  - The vertices V represent the parallelepipedic items or containers (via their id) of the arrangement,
  - If node *i* (attached to container *i*) and node *j* (attached to container *j*) are neighbors, we are sure that the real distance between the 2 nodes is less than $R_{2}$. If these two nodes are not neighbors, we are sure that the real distance between the 2 nodes is greater than $R_{1}$. The edges E of the graph represent this proximity information between the containers (an edge exists between vertices $V_{i}$ and $V_{j}$, if the two nodes are neighbors, and otherwise do not exist).

With these data and these constraints, is it possible to find how the containers are arranged within the composite container? This is the simple question addressed here in this paper.

### 3.2. Instrumentation Influence

As presented in the introduction, each container is instrumented by a node in charge of the data generation to obtain the neighborhood graph. The node is placed on one of the vertices of the parallelepiped. The placement of this node gives rise to particular constraints on the orientations of the container in the space, which ultimately influence the placement possibilities of the container. We first present this aspect of our study.

**Figure 2:**
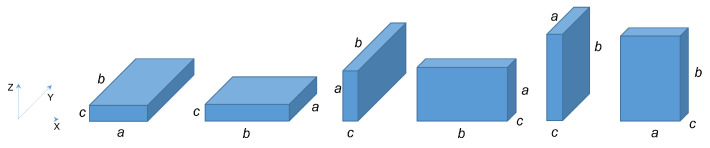
The possible space occupations of a parallelepiped in the 3D space.

A parallelepiped container of dimensions a×b×c (with a≠b≠c) with its edges parallel to the three axis of a Cartesian coordinate system can occupy 6 different volumes in the 3D space [25]. Figure 2 illustrates these 6 different cases.

**Figure 3:**
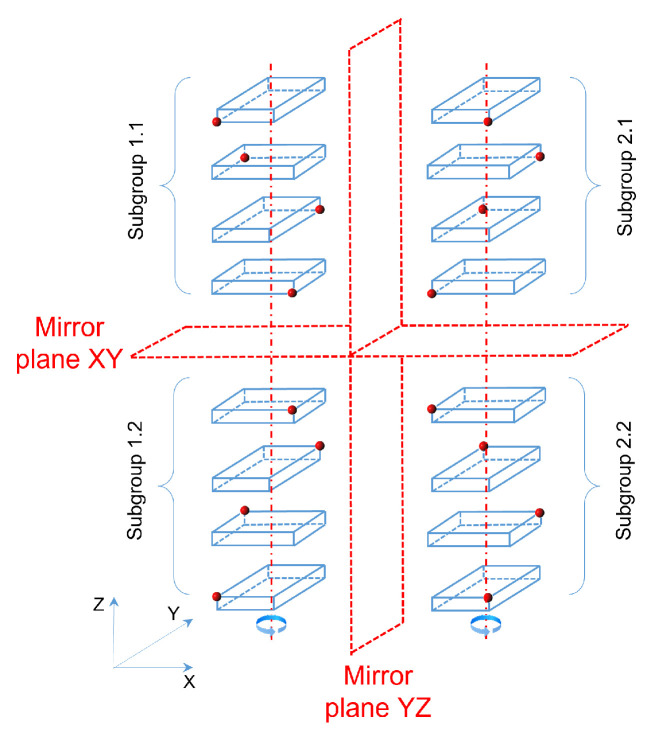
Impact of instrumentation on the combinatory of the problem due to the position of the node on the container.

The same parallelepipedic container fitted with a node on one of its vertices becomes asymmetric with respect to the different planes of the fixed reference frame. The position of the node relative to the volume must then be considered. Let us consider, for example, the case where the face (a×b) of the parallelepiped is parallel to the plan XY of a Cartesian coordinate system (that is 2/6 of the possible situations—cases 1 and 2 on Figure 2). In this situation 16 possibilities of orientation in the space can appear (as shown Figure 3) and 48 possibilities (48=3×16) if the supports are situated on the two other types of side. The 16 possible figures can be divided into 4 subgroups. In each subgroup, a rotation of $90^{\circ }\times k,\left(k\in \left\{0,1,2,3\right\}\right)$ around the z axis makes it possible to pass from one figure to another.

The asymmetry (mirror effect) between the subgroups 1.1 and 2.1, 1.2 and 2.2 with regard to the plan YZ, and between the subgroups 1.1 and 1.2, 2.1 and 2.2 with regard to the plan XY (Figure 3), makes impossible the passage by diverse rotations of any figure of one of these subgroups to those of another one: the figures are chiral. This is bound to their instrumentation, which can be realized by two different manners.

On the same figure, we can also notice the possibility of obtaining the figures of the subgroup 2.2 from those of the subgroup 1.1 (or conversely) by combinations of rotations around axes—idem between the subgroups 1.2 and 2.1: figures are achiral between these subgroups. We shall notice in these cases an inversion of the high/low position of the node in the space between these subgroups. Thus, there exist, for a parallelepiped instrumented on one of his summits, 8 possibilities of orientations in the space for each type of bearing surface (union of subgroup 1.1 and 2.2 or union of subgroup 1.2 and 2.1). The reduction of the 48 placement possibilities to be tested can be achieved thanks to an accelerometer (very low-cost solution) placed in the node (thus at an origin point) and whose axes are aligned on the axes of the container. The delivered information then allow:

- To determine the type of bearing face (face of dimension a×b, or a×c, or b×c) because of its perpendicularity with the axis *z* of the accelerometer (from 48 to 16 possibilities),
- To determine for each type of bearing surface the respective dimensions of the container along the x and y axis, thanks to the alignment achieved (from 16 to 8 possibilities),
- To know the node position on the container by taking into account the acceleration direction according to z (positive or negative) (from 8 to 4 possibilities).

This information ultimately allows, for an instrumented container, to test only the 4 solutions that appear in a same subgroup, out of the 48 initial and potential solutions. We can note that the introduction of a compass in the node could allow us to know the orientation of the parallelepiped with regard to the fixed mark (only one possibility of placement). However, and contrary to an accelerometer, this type of sensor is very sensitive to magnetic fields of the environment. This type of information was not thus taken into account. Thus, in the algorithm that we propose, 4 possibilities of placement must be tested for each of the containers if we take into account the positioning of the node on the box (chirality) and the orientation information of the vertical axis obtained by an ad hoc instrumentation.

### 3.3. Proposed Model

Parameters:

- *n*: number of unitary π-containers $\pi c_{i}$ with i∈{1,…,n} stacked in the composite π-container $\pi c_{0}$. This number *n* can evolve during the time and can be determine by a counting algorithm (no presented here).
- $\left(a_{i},b_{i},h_{i}\right)$: Non-negative external dimensions indicating the parallelepiped base dimensions (bearing face $a_{i}\times b_{i}$) and height $h_{i}$ of the π-container $\pi c_{i}$ (expressed here as integers).
- $O_{i}$: Binary parameter set when setting the node on the box.
  - If $O_{i}=0$, then the node is below the container;
  - Otherwise, the node is above.
- sd: A standard size expressed in meter.
- $\left(A_{0},B_{0},H_{0}\right)$: Non-negative external dimensions indicating the parallelepiped base dimensions (bearing face $A_{0}\times B_{0}$) and height $H_{0}$ of the composite π-container $\pi c_{0}$ (expressed here as integers).
- $R_{1}$: Transmission range (low threshold level) of sensor nodes. $R_{1}$ is dimensionless (to obtain this distance in meter, $R_{1}$ should be multiplied by sd).
- $R_{2}$: Transmission range (high threshold level) of sensor nodes. $R_{2}$ is dimensionless (to obtain this distance in meter, $R_{2}$ should be multiplied by sd).
- *V*: i×j matrix with *i* and j∈{0,…,n}. The matrix elements $v_{i,j}$ denote the neighbor relationship between containers $\pi c_{i}$ and $\pi c_{j}$.
  - $v_{i,j}=v_{j,i}=1$ (with i≠j), if distance $\left(\pi c_{i},\pi c_{j}\right)\leq R_{2}$
  - $v_{i,j}=v_{j,i}=0$ (with i≠j), if distance $\left(\pi c_{i},\pi c_{j}\right)>R_{1}$

These elements are deduced from proximity measurements between containers.

Variables;

- $\left(x_{i},y_{i},z_{i}\right)$: Discrete integer variables indicating the coordinates of the FLB (Front-Left-Behind) corner (the closest to the marker’s origin) of container $\pi c_{i}$.
- $\left(xn_{i},yn_{i},zn_{i}\right)$: Discrete integer variables indicating the coordinates of the sensor node fixed to container $\pi c_{i}$.
- $(Right_{i,j}$, $Left_{i,j}$, $Behind_{i,j}$, $Front_{i,j}$, $Below_{i,j}$, $Above_{i,j})$: Set of six binary variables that define the relative positions in the composite π-container of each pair of π-containers $\left(\pi c_{i},\pi c_{j}\right)$. Binary variables will be 1 if the container $\pi c_{j}$ is to the right of, to the left of, behind, in front of, below, or above the container $\pi c_{i}$, respectively; otherwise, 0. These variables will be used to ensure that containers do not overlap.
- $s_{i}$: Binary variable equal to 1 if the side of length $a_{i}$ of box number *i* is parallel to the side of length $A_{0}$ of the composite π−container, otherwise it is equal to 0.
- $t_{1,i},t_{2,i},t_{3,i},t_{4,i}$: Binary variables who represent the four possible relations between the coordinates of FLB corners and the coordinates of the sensor node of a container (see constraints 11a, 11b, 11c).

Note: It can be noted here that the variables $s_{i}$ and the variables $t_{1,i},t_{2,i},t_{3,i},t_{4,i}$ are linked. Indeed, when $t_{1,i}=1$ or $t_{3,i}=1$, then $s_{i}=1$, and $s_{i}=0$ when $t_{2,i}=1$ or $t_{4,i}=1$, hence $s_{i}=t_{1,i}+t_{3,i}$. The proposed model keeps variables $s_{i}$ to facilitate its understanding.

From the parameters and variables described above, the constraints of the CSP problem are as follows:

(1) $$\forall i,j,1\leq i,j\leq n:Right_{i,j}\left[x_{j}-\left(x_{i}+s_{i}a_{i}+\left(1-s_{i}\right)b_{i}\right)\right]\geq 0$$

(2) $$\forall i,j,1\leq i,j\leq n:Left_{i,j}\left[x_{i}-\left(x_{j}+s_{j}a_{j}+\left(1-s_{j}\right)b_{j}\right)\right]\geq 0$$

(3) $$\forall i,j,1\leq i,j\leq n:Behind_{i,j}\left[y_{j}-\left(y_{i}+s_{i}b_{i}+\left(1-s_{i}\right)a_{i}\right)\right]\geq 0$$

(4) $$\forall i,j,1\leq i,j\leq n:Front_{i,j}\left[y_{i}-\left(y_{j}+s_{j}b_{j}+\left(1-s_{j}\right)a_{j}\right)\right]\geq 0$$

(5) $$\forall i,j,1\leq i,j\leq n:Below_{i,j}\left[z_{i}-\left(z_{j}+h_{j}\right)\right]\geq 0$$

(6) $$\forall i,j,1\leq i,j\leq n:Above_{i,j}\left[z_{j}-\left(z_{i}+h_{i}\right)\right]\geq 0$$

(7) $$\forall i,j,1\leq i,j\leq n:Right_{i,j}+Left_{i,j}+Behind_{i,j}+Front_{i,j}+Below_{i,j}+Above_{i,j}\geq 1$$

(8) $$\forall i,1\leq i\leq n:x_{i}+s_{i}a_{i}+\left(1-s_{i}\right)b_{i}\leq A_{0}$$

(9) $$\forall i,1\leq i\leq n:y_{i}+s_{i}b_{i}+\left(1-s_{i}\right)a_{i}\leq B_{0}$$

(10) $$\forall i,1\leq i\leq n:z_{i}+h_{i}\leq H_{0}$$

(11) $$\forall \left\{i|1\leq i\leq n\right\}:\left(xn_{i},yn_{i},zn_{i}\right)\in \left\{\left(x_{i},y_{i},z_{i}+O_{i}.h_{i}\right),\left(x_{i},y_{i}+a_{i},z_{i}+O_{i}.h_{i}\right),\left(x_{i}+a_{i},y_{i}+b_{i},z_{i}+O_{i}.h_{i}\right),\left(x_{i}+b_{i},y_{i},z_{i}+O_{i}.h_{i}\right)\right\}$$

(11) can also be represented as:

(11a) $$\begin{array}{c} \forall i,1\leq i\leq n:xn_{i}=t_{1,i}x_{i}+t_{2,i}x_{i}+t_{3,i}\left(x_{i}+a_{i}\right)+t_{4,i}\left(x_{i}+b_{i}\right) \end{array}$$

(11b) $$\begin{array}{c} \forall i,1\leq i\leq n:yn_{i}=t_{1,i}y_{i}+t_{2,i}\left(y_{i}+a_{i}\right)+t_{3,i}\left(y_{i}+b_{i}\right)+t_{4,i}y_{i} \end{array}$$

(11c) $$\begin{array}{c} \forall i,1\leq i\leq n:t_{1,i}+t_{2,i}+t_{3,i}+t_{4,i}=1 \end{array}$$

(11d) $$\begin{array}{c} \forall i,1\leq i\leq n:zn_{i}=z_{i}+O_{i}.h_{i} \end{array}$$

(12) $$\forall i,j,0\leq i,j\leq n:v_{i,j}\sqrt{{\left(xn_{i}-xn_{j}\right)}^{2}+{\left(yn_{i}-yn_{j}\right)}^{2}+{\left(z_{i}-z_{j}\right)}^{2}))}\leq v_{i,j}R_{2}$$

(13) $$\forall i,j,0\leq i,j\leq n:\left(1-v_{i,j}\right)\sqrt{\left(\left({\left(xn_{i}-xn_{j}\right)}^{2}+{\left(yn_{i}-yn_{j}\right)}^{2}+{\left(z_{i}-z_{j}\right)}^{2}\right)\right)}>\left(1-v_{i,j}\right)R_{1}$$

**Figure 4:**
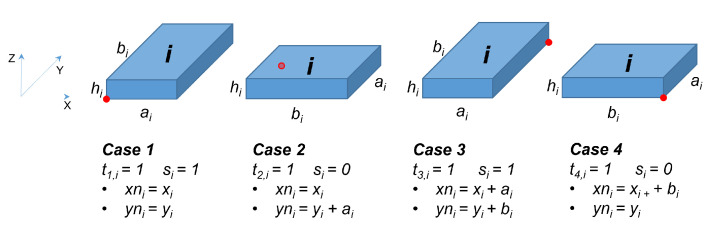
Links between FLB corner and node position.

Constraints (1)–(7) are non-overlapping conditions and certify that π-containers do not overlap whatever their orientation. Constraints (8)–(10) ensure that all π-containers are within the composite π-container. These two first sets of constraints are classic in a CLP model. Constraints (11) represent the relation between the coordinates of the FLB corner and of the sensor node. The sensor node can potentially be placed at one of the four corners of the π-container according to π-containers can be freely rotated. The implementation of these n constraints is carried out by means of the constraints 11a, 11b, 11c and 11d. Figure 4 graphically represents these 4 possibilities for container i. Constraints (12) and (13) certify that all neighborhood (and non-neighborhood) relationships have been respected. This last set of constraints, (11)–(13), is totally new. It represents the specificities of the problem presented in this paper. This model leads us to find the absolute coordinates and the orientation of each π-container, which satisfy the neighbor relationships between the sensor nodes.

### 3.4. Proximity Graph—Generalization to Cases of Several Measurements

The model presented above can be generalized in case *m* times of container proximity measurements with *m* different coverage radius are repeated. Thus, it would be possible to obtain *m* proximity graphs as a function of the *m* values of ($R_{1}$, $R_{2}$) used for the measurements. Constraints (12) and (13) of the model would thus be instantiated *m* times. We propose here to aggregate these *m* instances in the model.

Let $d_{ij}$ be the real distance between 2 distinct $\pi c_{i}$ and $\pi c_{j}$ nodes. As we have already presented in our initial model, we have:

$$v_{i,j}=v_{j,i}=1\left(\mathrm{with}\;i\neq j\right),\mathrm{if}\;\mathrm{distance}\;\left(\pi c_{i},\pi c_{j}\right)\leq R_{2}$$

$\pi c_{i}$ and $\pi c_{j}$ are neighbor

$$v_{i,j}=v_{j,i}=0\left(\mathrm{with}\;i\neq j\right),\mathrm{if}\;\mathrm{distance}\;\left(\pi c_{i},\pi c_{j}\right)>R_{1}$$

$\pi c_{i}$ and $\pi c_{j}$ are not neighbors.

By performing *m* measurements with $R_{1}^{k}$ and $R_{2}^{k}$ as bounds of the kth measurement (∀k∈[1,m]) and $R_{1}^{k}\leq R_{2}^{k}$, we have:

$$\mathrm{if}\;v_{ij}^{k}=1\;\mathrm{then}\;d_{ij}^{k}\in [0,R_{2}^{k}]\;\mathrm{else}\;,d_{ij}^{k}\in \left[R_{1}^{k},+\infty \right]$$

and we can deduct from it by merging the *m* intervals, and thus find that: $d_{ij}\in \left[\max_{k}R_{1}^{2}k,\min_{k}R_{2}^{k}\right]$. Equations (12) and (13) can therefore be written in the form (generalization to *m* measurements):   

(14) $$\forall i,j,0\leq i,j\leq n,k\in \left[1,m\right]:v_{i,j}\sqrt{{\left(xn_{i}-xn_{j}\right)}^{2}+{\left(yn_{i}-yn_{j}\right)}^{2}+{\left(zn_{i}-zn_{j}\right)}^{2})}\leq v_{i,j}.\min_{k}R_{2}^{k}$$

(15) $$\forall i,j,0\leq i,j\leq n,k\in \left[1,m\right]:\left(1-v_{i,j}\right)\sqrt{{\left(xn_{i}-xn_{j}\right)}^{2}+{\left(yn_{i}-yn_{j}\right)}^{2}+{\left(zn_{i}-zn_{j}\right)}^{2}}\leq \left(1-v_{i,j}\right).\max_{k}R_{1}^{k}$$

The purpose of taking into account *m* constraints of proximity in this form is to, if necessary, reduce the number of potential solutions to the problem initially posed.

### 3.5. Implemented Algorithm

The proposed algorithm (Algorithm 1) counts and enumerates all the solutions of this Constraints Satisfaction Problem. It proposes in output all the solutions respecting all the constraints.
**Algorithm 1** Developed algorithm1:**Function** Solve2:    ListSolutions clear3:    **GatewaysPlacement**4:    **FixedContainersPlacement()**                   // if we know the exact position of certain items (optional)5:    **ContainerPlacement(1)**                         //recursive call6:    **foreach** solution S in ListSolutions7:      **if** CheckSolutionStaticEquilibrum(S) = false **then**8:         ListSolutions remove(S)9:**Function** ContainerPlacement(i : ContainerNumber)10:    **foreach** values $s_{i},x_{i},y_{i},z_{i}$ satisfying constraints (1) to (10) with previous placed containers                   // containers geometric placement is OK without overlapping11:      **foreach** values tn,i satisfiying constraints (11)                   // gots all xni, yni, zni location of the sensor network from $x_{i},y_{i},z_{i}$12:         **if** (xni, yni, zni) is satisfiying constraints (12) (13) **then**                   // (xni, yni, zni respects the neighborhood graph)13:           **if** i = lastcontainernumber **then**14:              ListSolutions add current15:           **else**16:              **ContainerPlacement** (i+1)

This algorithm enumerates in a first time the set of solutions that respect all the constraints previously presented. A verification of each of the generated solutions is a posteriori performed to verify the static balance of the whole. Naturally, and for the sake of efficiency, the largest containers are placed first. The algorithm shows the GatewaysPlacement function. In the context of our paper, this function places a single node connected to the container $\pi c_{0}$. The position of this node serves as the spatial reference or origin of the Cartesian system in which we want to identify the positions of all the containers. This function also makes it possible to insert in the network additional WSN nodes (but not necessarily) not linked to physical containers, but whose position in space is known. These nodes support communications to the outside of the system, and potentially introduce additional proximity constraints that can reduce the number of ultimately generated solutions.

## 4. Test and Discussion

### 4.1. Introduction and Presentation of Cases

**Figure 5:**
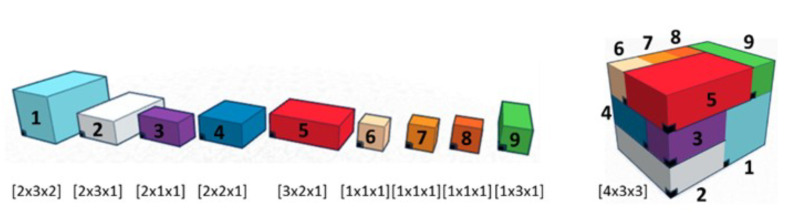
Meller’s scenario with containers and composite container.

**Figure 6:**
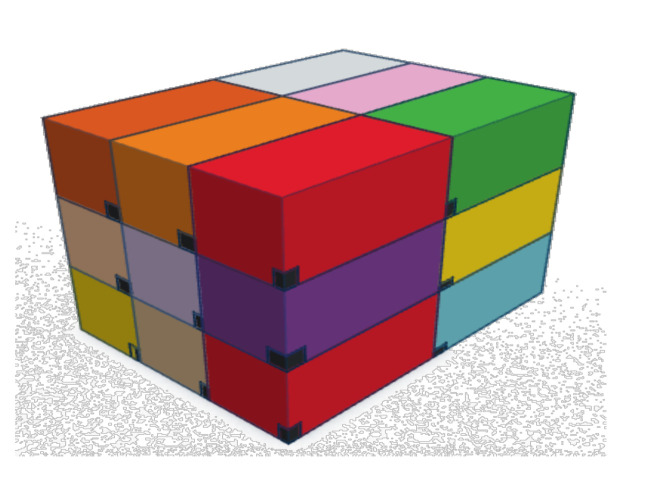
Arrangement of 18 containers with identical dimensions.

Here, the objectives are, in the first instance, to verify and validate the proposed model, but also, on the basis of an enumeration of the solutions, to study the simple possibilities of reducing their number. To do this, we will rely on three different scenarios of arrangement. The first one is a classic case of literature. This case, named Meller’s scenario [26], is shown Figure 5. It consists of nine containers of different dimensions where WSN nodes position is represented in black. This case is a widely used example in the Physical Internet community for various purposes, which is why we chose it. As our approach is completely new, there are no cases in the literature on which we can compare our results. This case was chosen because it presents containers of heterogeneous dimensions. The two other scenarios will be homogeneous cases consisting of 18 and 36 containers with identical dimensions (see Figure 6 for scenario with 18 containers). These two cases seem *a priori* less interesting from an application point of view, in particular if all containers are identical and embed the same products. However, the regularity of the container dimensions, or the homogeneity of the arrangements, is an interesting case to test the performance of our model.

For these three scenarios and for all the tests performed, we introduce here the notion of uncertainty (noted *I*) on the neighborhood measurement with respect to a coverage radius R from the values of $R_{1}$ and $R_{2}$ in the form:

$R_{1}=R-I$

$R_{2}=R+I$

This change in notation does not change the proposed model in any way.

### 4.2. Tests and Results without Measurement Error

**Figure 7:**
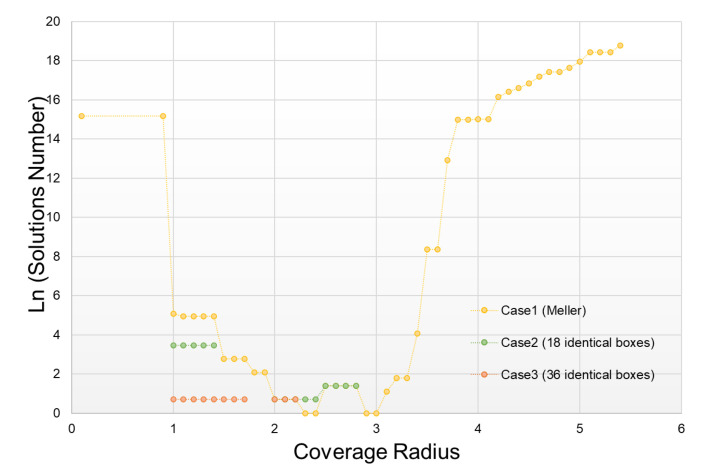
Enumeration of solutions according to the coverage radius (without uncertainty).

The first results concern the enumeration of the solutions obtained in the three test cases mentioned above, considering that the measurement uncertainty is zero (I=0 or $R=R_{1}=R_{2}$) and with one single radius (no repetition of measurements, or m = 1). Different coverage radius has been tested over a range from 0.1 to 5.4 (length of the composite container diagonal), with a spatial sampling of 0.1. The results obtained are shown Figure 7 with a logarithmic scale. This confirms our initial results (see [5]) and shows the very strong influence of *R* on the number of solutions obtained. For low or high *R* values in relation to the relative dimensions of the containers, a combinatorial explosion can be observed: the containers either have no neighbors or all the containers are all neighbors. In these two cases, this information does not contribute anything, and neighborhood constraints do not in any way reduce the number of solutions obtained. In the Meller’s scenario, it is possible to find the real arrangement (only 1 solution for values of *R* = 2.3, *R* = 2.4, *R* = 2.9, *R* = 3.0) whereas in cases 2 and 3, at least 2 solutions can be established. The heterogeneity of the initial arrangement is an advantage in the approach we propose.

In order to situate the quality of our results, we tried to establish the number of solutions we could obtain with a perfect knowledge of the distances separating each container (all known $d_{ij}$). This number of solutions can be considered as the lower bound of the problem as we posed it. To do this, we used the same model and the same algorithm as previously presented by taking, for each pair $\left(\pi c_{i},\pi c_{j}\right)$ of the arrangement, a measure *k* where: $\max_{k}R_{1}^{k}=\min_{k}R_{2}^{k}$. In other words, we know the distance $d_{ij}$ between each container.

This enumeration of solutions was carried out for each of the 3 cases studied here. The results obtained are showed in Table 1.

These tests show us that in certain situations, and with much more information than proximity, it is impossible to find one and only one solution (only one twin!). On the other hand, as the number of solutions is not very high, it is possible to differentiate them by other means.

### 4.3. Tests and Results Taking into Account Measurement Errors

**Figure 8:**
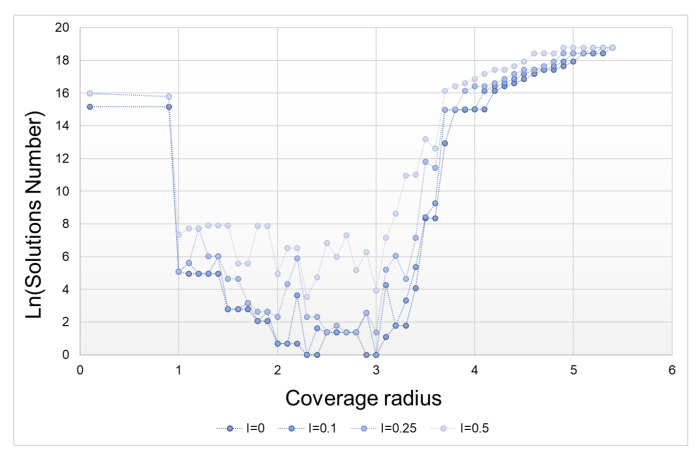
Influence of the uncertainty on the number of solutions according coverage radius (Meller’s scenario).

The second type of tests that we then conducted allowed us to quantify the influence of the measurement uncertainty on the solutions number obtained in each of the three cases. Figure 8 shows the results obtained for the Meller’s scenario. Not surprisingly, as uncertainty increases, so does the number of solutions. The minimum number of solutions is, for this case, to be found in a range of *R* between 2 and 3.

The same study was conducted for cases 2 and 3. The results are presented in Table 2 and Table 3. Since the number of solutions found for some *R* values was enormous, we limited the runtime of each enumeration to 5 min or to a maximum of 1000 values (situations represented by a “-” in the tables).

The results obtained on the three cases, show the difficulty to obtain a very limited number of solutions if the uncertainty increases, but also to fix the value of R, which seems to depend on the dimensions of the containers.

### 4.4. Tests and Results with Additional Neighborhood Constraints

**Figure 9:**
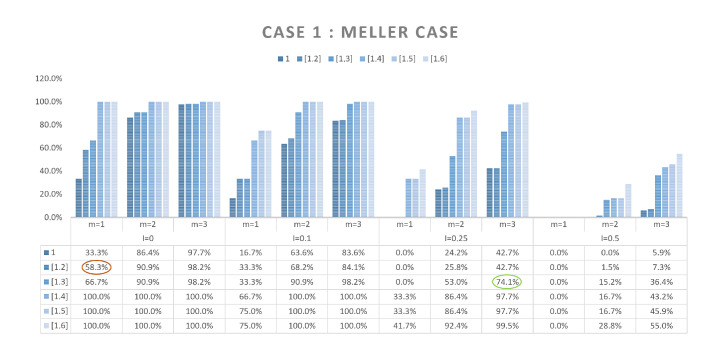
Histogram of the solutions number per range—Meller’s scenario.

**Figure 10:**
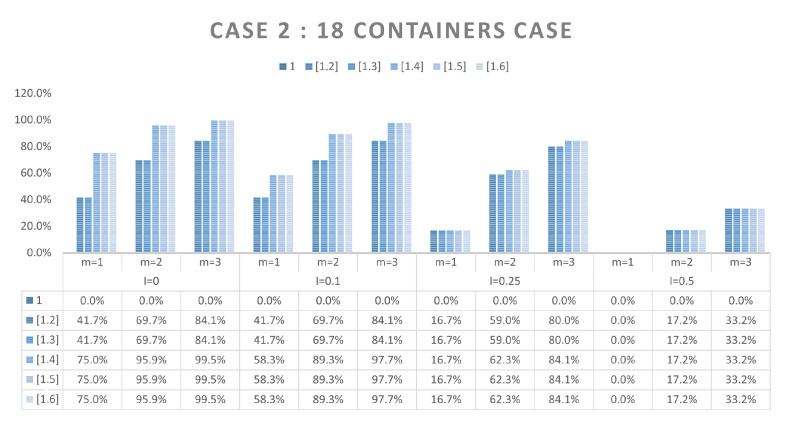
Histogram of the solutions number per range—18 containers scenario.

In order to reduce the number of solutions obtained, we propose here to add additional constraints for the resolution of the problem. Thus, we will consider that the neighborhood measurements carried out are repeated (constraints 12 and 13 of the proposed model) with different values of $R_{1}$ and $R_{2}$ (constraints 14 and 15). We will compare the results obtained with a single measurement (m=1) with those obtained for m=2 and m=3. We have limited ourselves to 3 different radii, because these measurements have a cost in terms of time, but also because the combinatorics of the cases to be tested became too important. These tests were carried out on scenarios 1 and 2, with 9 and 18 containers respectively and *R* values between 2 and 3.1 (zone of interest obtained for these 2 cases). *R* varies with a step of 0.1, which gives us in the end 12 cases to test for m=1, 66 cases for m=2, and 220 cases for m=3, (i.e., the number of combinations of 2 or 3 radius in the 12 possible radius). Finally, these different tests were repeated for uncertainty *I* values of 0, 0.1, 0.25 and 0.5. The results obtained are summarized Figure 9 and Figure 10. The two histograms illustrate the percentage of the number of solutions found for a given range in relation to the totality of solutions tested, and this for each of the uncertainties with m=1, m=2 and m=3. For example, the value of 58.3% (red circle in the figure) indicates that for an uncertainty of 0, with m=1, 58.3% of all cases tested (i.e., 7 cases out of the 12 tested for a single radius) find one or two solutions. The value of 74.1% (green circle in figure) indicates that for an uncertainty at 0.25, with m=3, 74.1% of all cases tested (163 cases out of the 220 tested for 3 radius) find one to three solutions. One can thus visualize the effect of taking into account several radii on the possibilities of obtaining a smaller quantity of solutions. In both cases, the influence of taking these additional constraints into account is very clear, even in the case where the uncertainty is the highest (I=0.5). Nevertheless, there are still possibilities to obtain several solutions, and thus do not find the digital twin of the real arrangement.

## 5. Conclusions and Perspectives

In this paper, we have proposed a new type of packing problem. This problem aims at obtaining the digital twin of a composite container independently of time and location, thanks to a network of sensors linked to the items contained in the container. The solution recommended and tested in this paper is based on neighborhood (or proximity) information obtained by the sensor network.

This problem has allowed us to propose a mathematical model taking into account these proximity constraints, but also the particular constraints generated by the instrumentation. On these bases, an algorithm was developed in order to validate this model and to enumerate the solutions obtained on various cases of arrangement, with broad ranges of coverage radius, various measurement uncertainties, as well as the repetition of measurements of proximity with various coverage radii. The obtained results validate our model and show that the use of several coverage radii is necessary to obtain a limited number of eligible solutions as a digital twin, especially when the measurement uncertainty becomes large.

Both the original proposed application (numerical twin of a container) and the proposed model are new contributions to the research. This model has similarities, but also differences with the models classically used in the initial construction of an arrangement in a container.

The current limitations of the work presented here lie mainly in the number of potentially eligible twins, and thus in the difficulty of retrieving the physical container arrangement. To remedy this, in other words to reduce the solution space, adding additional constraints to the model is the most natural solution path. We have partially tested it by repeating proximity measurement campaigns. To go further, it seems that other constraints, based on measurements of different nature (contact between items for example), can lead to the obtaining of this container-DT.

## Figures and Tables

**Table 1:** Enumeration of the solutions found with a perfect knowledge of the distances $d_{ij}$ between each container. (The “-” in the table are linked either to a timeout (fixed at 5 minutes) or to a number of solutions greater than 1000).

Scenarios			
Uncertainty I	Meller	18 Containers (1 × 1 × 2)	36 Containers (1 × 1 × 1)
0	1	2	2
0.1	1	2	2
0.25	1	2	2
0.5	1	2	2
0.75	1	2	2
1	1	2	-
2	32	-	-

**Table 2:** Influence of the uncertainty on the number of solutions obtained with different coverage radius—scenario 2.

	Number of Solutions						
***R***	**I = 0**	**I = 0.1**	**I = 0.25**	***R***	**I = 0**	**I = 0.1**	**I = 0.25**
<1	-	-	-	1.9	24	-	-
1	32	32	32	2	2	2	2
1.1	32	288	288	2.1	2	2	2
1.2	32	32	-	2.2	2	2	-
1.3	32	32	-	2.3	2	2	-
1.4	32	-	-	2.4	2	2	-
1.5	32	-	-	2.5	4	-	-
1.6	32	32	-	2.6	4	4	-
1.7	32	-	-	2.7	4	4	-
1.8	24	-	-	2.8	4	-	-
				>2.8	-	-	-

**Table 3:** Influence of the uncertainty on the number of solutions obtained with different coverage radius—scenario 3.

	Number of Solutions						
**R**	**I = 0**	**I = 0.1**	**I = 0.25**	**R**	**I = 0**	**I = 0.1**	**I = 0.25**
<1	-	-	-	1.6	-	-	-
1	2	2	2	1.7	-	-	-
1.1	2	2	2	1.8	-	-	-
1.2	2	2	-	1.9	-	-	-
1.3	2	2	-	2	2	-	-
1.4	2	-	-	2.1	2	-	-
1.5	-	-	-	2.2	2	-	-
				>2.2	-	-	-

## Data Availability

No new data were created or analyzed in this study. Data sharing is not applicable to this article.
